# Biological network analysis with CentiScaPe: centralities and experimental dataset integration

**DOI:** 10.12688/f1000research.4477.2

**Published:** 2015-07-07

**Authors:** Giovanni Scardoni, Gabriele Tosadori, Mohammed Faizan, Fausto Spoto, Franco Fabbri, Carlo Laudanna

**Affiliations:** 1 Center for Biomedical Computing, University of Verona, Verona, 37134, Italy; 2 Birla Institute of Technology & Science, Goa, 403726, India; 3 Department of Computer Science, University of Verona, Verona, 37134, Italy; 4 Department of Pathology and Diagnostics, University of Verona, Verona, 37134, Italy

**Keywords:** Cytoscape, centrality indexes, nodes, networks, topological analysis

## Abstract

The growing dimension and complexity of the available experimental data generating biological networks have increased the need for tools that help in categorizing nodes by their topological relevance. Here we present CentiScaPe, a Cytoscape app specifically designed to calculate centrality indexes used for the identification of the most important nodes in a network. CentiScaPe is a comprehensive suite of algorithms dedicated to network nodes centrality analysis, computing several centralities for undirected, directed and weighted networks. The results of the topological analysis can be integrated with data set from lab experiments, like expression or phosphorylation levels for each protein represented in the network. Our app opens new perspectives in the analysis of biological networks, since the integration of topological analysis with lab experimental data enhance the predictive power of the bioinformatics analysis.

## Introduction

Biological processes can be abstracted as networks where the nodes represent biological entities and the edges represent interactions between them. Several kind of biological networks have been described: metabolic networks, gene networks, signal transduction networks and protein-protein interaction networks ^1^. Such networks are a static representation of the dynamics of complex biological processes, where molecular interactions give rise to cascades of reactions called pathways, determining the life processes of living organisms. Even if the scientific community is far from the capability of simulating the dynamical behaviour of such pathways, several important information can be extracted from the topological analysis of biological networks ^2, 3^ since the structure of a network can affect its function ^4^. In this context, different global parameters are commonly used to describe the properties of the whole network: centralities ^5^ are indexes that permit the identification of the most important actors by characterizing the nodes that, more than others, are good candidates as regulators of the underlying biological process.

In this paper we present CentiScaPe 2.1, a Cytoscape app ^6, 7^ for network centralities analysis. While the built-in NetworkAnalyzer tool is oriented in characterizing the global behaviour of the network, provided with several global network statistics, CentiScaPe is designed to identify the most relevant nodes and provides a more comprehensive set of centralities. The new version introduces the computation of centralities for directed and weighted networks, two features that are not available in any other Cytoscape app. As in the previous version it computes Average Distance, Diameter, Degree, Stress, Betweenness, Radiality, Closeness, Centroid Value and Eccentricity. New indexes as Eigenvector, Bridging centrality and Edge Betweenness have been added, making it the most complete app for network centrality analysis. Some hypothesis about the centralities interpretation and application in a real biological context are presented in the Cen­tralitiesTutorial (see the Supplementary materials section).

A web version of CentiScaPe, i.e. FastCentiScaPe, is also available ( http://www.cbmc.it/fastcent/). It performs very fast computations for large networks by sending the network to a multiprocessor server. The centrality analysis results are sent to the user by e-mail in a Cytoscape readable, i.e. *.xgmml*, format.

The main aim of CentiScaPe is to produce results that could drive further lab experiments since the high score nodes identified by the computation should be considered as potential targets for drugs and could be the starting point for further investigations and for a deeper understanding of the underlying biological process.

## Methods and implementation

The main usage of CentiScaPe is to rank the nodes of a network depending on their topological and experimental relevance. The numerical results are saved as node, edge or network attributes in the Cytoscape attributes browser, depending on the kind of parameters, so all the Cytoscape features for managing attributes are supported; after the computation the centralities are treated as normal Cytoscape attributes. CentiScaPe can be used in undirected networks ^8^, in directed networks and in weighted networks. Centralities for directed networks (see Supplementary materials: CentralitiesTutorial) are useful in the case of metabolic networks in which the direction describes the interaction between the substrates and reactants and the products of the chemical reactions and also in signal transduction networks, in which the direction depends on the flux of information. Considering the direction in the computation of centralities can lead to different results and interpretations than the undirected version.

**Figure 1.**
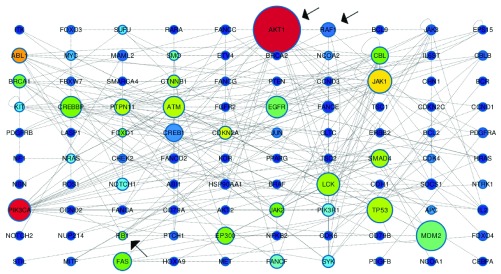
Stress computed on oncogenes. Size represents directed Stress, colour undirected Stress (blue=low, red=high).

As an example, in Figure 1 we show the computation of the directed and undirected Stress applied to a network of Oncogenes (see Supplementary materials: Oncogenes.txt and Oncogenes_edge_directions.txt). Results of both the computations are shown and discussed.

The image, obtained using Cytoscape’s graphical tool, represents the different Stress values by using the colour and the size of the nodes. The size describes the value obtained by using the directed Stress: the bigger the node the higher the value; the colour describes the value obtained by using the undirected Stress: red is used for the highest value, blue for the lowest value. For example a large blue node requires particular attention because it is showing a node with a high centrality value if the network is considered as directed but with a very low value if the network is considered as undirected.

By analyzing the Oncogenes network we saw that the large red node, i.e. AKT1, shows how its Stress values are high using both algorithms. It plays a central role in different cell processes like metabolism, proliferation, cell survival, growth and angiogenesis. This role may highlight its high Stress value but, on the other hand, the high values suggest us to deeply investigate its characteristics; it is also involved in two different kind of cancer: breast and colorectal (see http://www.uniprot.org/uniprot/P31749). This evidence suggest that the node could be involved in cancer related processes but this assertion needs validation from several lab experiments.

Another interesting result is shown by the blue medium sized node RAF1. It shows how, using undirected Stress we obtained a low value, but by using the new algorithm we obtained an interesting Stress value. RAF1 was identified as a proto-oncogene with different and fundamental cellular functions (see http://www.omim.org/entry/164760). The results could be interpreted by saying that the directed network gives us a better understanding about how the gene, and its product, are involved in the development of cancer and could highlight that the use of the direction enhance our ability in describing a complex biological process.

The opposite situation is found in the third highlighted node, the small green node, RB1 in the right bottom corner. In this case the value computed with undirected Stress is not very high, but the value computed with the directed Stress is very low. RB1 is a gene involved in coding a protein involved in the retinoblastoma and other type of cancer like bladder cancer and osteogenic sarcoma. It was the first tumor suppressor gene found (see http://www.ncbi.nlm.nih.gov/gene/5925). As already said for RAF1, if the directed analysis is considered more reliable than the undirected one, then RB1 seems to be marginally involved in the Oncogenes network otherwise the undirected network is a better description. As already stated an experimental validation should be carried out in order to improve the results from the topological analysis and to better understand the role of each highlighted node.

All the results shown and described must be considered as a possible direction for further lab experiments. The main goal of this kind of analysis is to give us a comprehensive view that could be useful in describing the role of each node involved in a specific biological process and to drive future insights and investigations.

Second important features of the new version of CentiScaPe is the possibility of computing centralities for weighted networks, that are networks in which the edges are provided with an attribute that can be interpreted as a distance between the two connected nodes.

**Figure 2.**
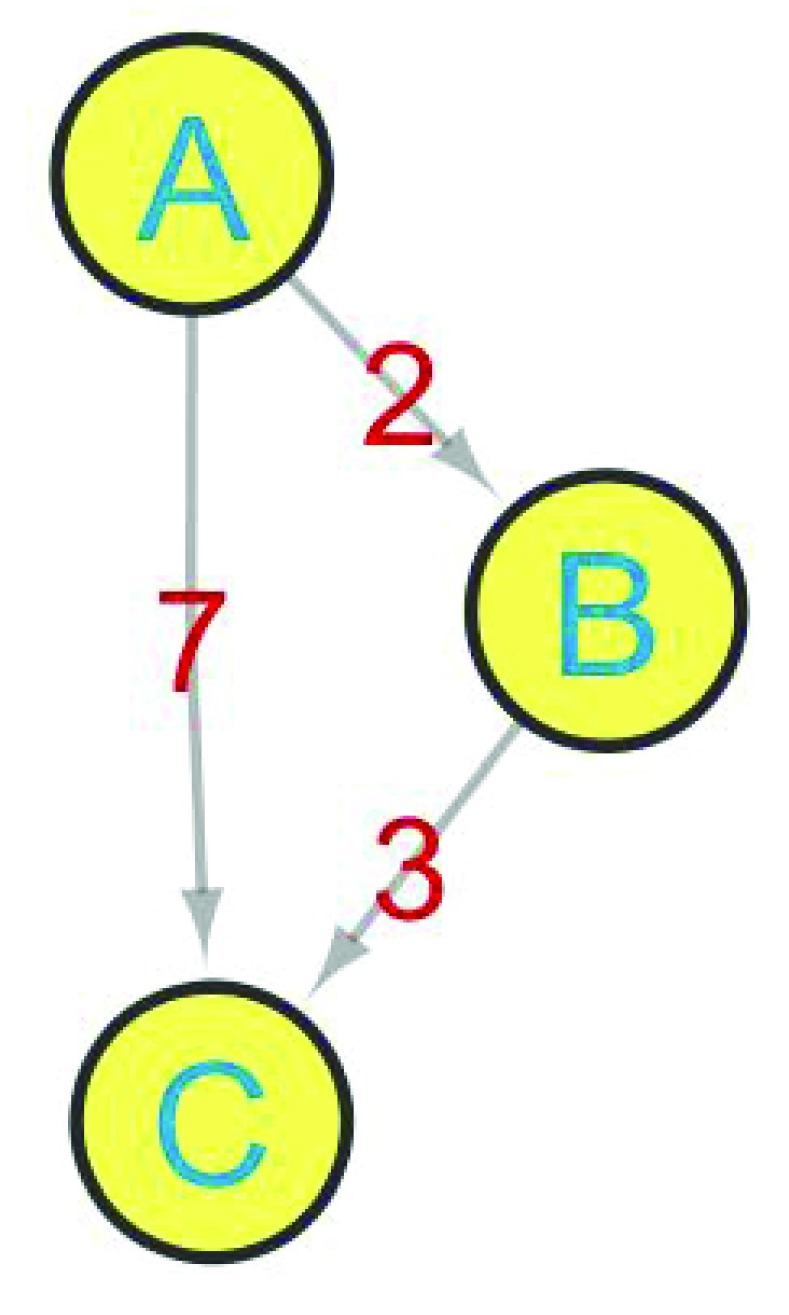
A weighted network. If the weight of the edge is not considered the shortest path from A to C is the directed edge, and the distance is one. If the weights are considered the shortest path from A to C is the one passing through B, and the distance is equals to 5.

In the network in Figure 2 we have a distance ( *dist*) attribute for each edge. The values are *dist*( *A*, *B*) = 2, *dist*( *B*, *C*) = 3 and *dist*( *A*, *C*) = 7. Since A and C are connected by a single edge, in an unweighted computation, the distance from A to C is equal to one. But if the attributes of the edges are considered as distances, the shortest path between A and C is the one passing through B (= 2 + 3 = 5) since it is shorter, or *lighter*, than the one connecting A directly to C (= 7). The computation of weighted shortest paths will result in completely different values from the case wherein the weight is not considered. The user should consider that the weight is used in the sense that close nodes are more important than distant nodes. Therefore depending on the meaning of the attributes, one can use the real value or its reciprocal. For example if the attribute represents the speed of a reaction instead of a distance, the reciprocal should be used. This is because the higher the value of the speed, the nearer the nodes are: an increasing speed determines a decreasing reciprocal and the distance decrease by consequence.

An example of usage of weighted networks centralities analysis, can be found in Holly *et al.* ^9^ where an euclidean distance is given to each edge depending on the difference between the phosphorylation level of the proteins connected by that edge.

All the graphical features of the previous version of CentiScaPe, as the plot-by-node, the plot-by-centrality and the boolean-based result panel have been maintained in the new version. A complete guide can be found in Scardoni *et al.* ^8^ or in the CentiScaPe userguide available from the website.

## Conclusions

CentiScaPe 2.1 have been enriched with new centrality parameters as Eigenvector, Bridging centrality and Edge Betweenness centrality and with the possibility to analyze directed and weighted networks. It allows integration centrality-based network analysis with experimental data by using nodes, edges or network attributes. The results of the computation can be used directly and are easily portable as Cytoscape attributes allowing the user to exploit all the other features of Cytoscape and its apps. Compared to the Cytoscape’s built-in tool, NetworkAnalyzer, CentiScaPe is an excellent integrative tool that allows the identification of potential target nodes from both the topological and the experimental point of view, and can be considered as an essential instrument for the characterizations of nodes in order to drive further experiments.

## Software availability

### Software available from the Cytoscape App Store

http://apps.cytoscape.org/apps/centiscape

### Latest source code

https://bitbucket.org/giovanniscardoni/centiscapepublic/

### Source code as at the time of publication

https://bitbucket.org/F1000Research/centiscapepublic-archive

### Archived source code as at the time of publication

http://dx.doi.org/10.5281/zenodo.10652 ^10^

### License

Lesser GNU Public License 3.0: https://www.gnu.org/licenses/lgpl.html

## Supplementary materials

Supplementary files available from:
