# Supplementary material for: Biological network analysis with CentiScaPe: centralities and experimental dataset integration
Source: F1000Res. 2015 Jul 7;3:139. Originally published 2014 Jul 1. [Version 2] doi: 10.12688/f1000research.4477.2 (PMC4647866; doi:10.12688/f1000research.4477.2)
Supplement: Supplementary file 1 [file f1000research-3-6690-s0000.tgz › dd4541d4-bdfe-461a-be92-f2e2cf9f2d57.zip!/supplementary_materials/CentralitiesTutorial.pdf]

CentiScaPe: Network centralities for Cytoscape

Giovanni Scardoni[1,*] Carlo Laudanna [1]
Gabriele Tosadori [1] Franco Fabbri [1]
Mohammed Faizaan[2]

[1] CBMC, University of Verona, [*] giovanni.scardoni@gmail.com,
[2] M.Sc.(Tech.) Information Systems BITS - Pilani, K. K. Birla Goa campus

Definitions

Let $G = (V, E)$ a directed or undirected graph, with $n = |V|$ vertexes. $deg(v)$, $deg(v)$, $deg+(v)$ indicate, respectively, the degree, the in-degree and the out-degree of the vertex. $dist(v, w)$ is the shortest path between v and w. σ_{st} is the number of shortest paths between s and t and $\sigma_{st}(v)$ is the number of shortest paths between s and t passing through the vertex v. Notably:

- Vertex = nodes; edges = arches;
- The distance between two nodes, $dist(v, w)$, always indicates the shortest path between the two nodes;
- All calculated scores are computed giving to higher values a positive meaning, where positive does refer to node proximity to other nodes. Thus, independently on the calculated node centrality, higher scores indicated proximity and lower scores indicate remoteness of a given node v from the other nodes in the graph G.

Undirected networks

Undirected networks are networks where there is no direction of the edges. The edges are considered as bi-directional and the classic centralities definition are applied.

Directed networks

Now centralities can be computed also for directed network. A directed network is a network where the edge has a direction. This means that given two nodes A and B, some times there are no paths betweens them, or there is a path from node A and B but not from node B to node A. This is very typical of signaling or metabolic networks, where informational or energy/mass flow follows a specific

direction determined by the thermodynamics of biochemical reactions. Some of the centralities definitions cannot be used in directed networks, so they have been redefined in order to consider direction of the edges.

Weighted networks

Centralities are based on the shortest path. In a weighted networks, the edges have an attribute value that is considered during the computation of the shortest-path. This value is used as a distance between nodes considered in the evaluation of the shortest paths between nodes. It means that if you have two paths connecting two nodes with the same number of edges but with different weights, only the shortest one will be considered, using the provided distance.

How to use edge weight

Suggestion 1: Provide the right values for the edge weight

Example
Suppose you have two paths connecting node A to node D. the paths are:

$$A \to B \to D$$

and

$$A \to C \to D$$

where the weights are the following:

$$w(A - B) = 1, w(B - D) = 1, w(A - C) = 2, w(C - D) = 1$$

Here the shortest path connecting A and D is $A - B - C$ and the total shortest path length is 2. The weight of the path $A - C - D$ is 3 and it is not considered, even if the number of edges of the two paths is the same.

Take care to consider the weight of the network in a proper way: If the weight means space or time it can be used, if the weight means speed you have to use $1/weight$ as attribute of the edge.

Suggestion 2: Provide values for the edge weight that are properly rounded

Example
Suppose you have two paths connecting node A to node D. the paths are:

$$A \to B \to D$$

and

$$A \to C \to D$$

where the weights are the following:

$$w(A - B) = 1, w(B - D) = 1, w(A - C) = 1,01, w(C - D) = 1$$

Here the shortest path connecting A and D is $A - B - C$ and the shortest path length is 2. The weight of the path $A - C - D$ is $2,01$ but it is not considered since the difference is very low. If you round the value of weight $A - C$ from $1,01$ to 1, the path $A - C - D$ is also considered, since its weight become 2 (the same of the other path).

Take care of consider the values properly rounded depending on the meaning of your edge attribute.

Centralities

Degree ($deg(k)$)

Is the simplest topological index, corresponding to the number of nodes adjacent to a given node v, where adjacent means directly connected. The nodes directly connected to a given node v are also called first neighbors of the given node. Thus, the degree also corresponds to the number of adjacent incident edges. In directed networks we distinguish in-degree, when the edges target the node v, and out-degree, when the edges target the adjacent neighbors of v. Calculation of the degree allows determining the degree distribution $P(k)$, which gives the probability that a selected node has exactly k links. $P(k)$ is obtained counting the number of nodes $N(k)$ with $k = 1, 2, 3 \ldots$ links and dividing by the total number of nodes N. Determining the degree distribution allows distinguishing different kind of graphs. For instance, a graph with a peaked degree distribution (Gaussian distribution) indicates that the system has a characteristic degree with no highly connected nodes. This is typical of random, non-natural, networks. By contrast, a power-law degree distribution indicates the presence of few nodes having a very high degree. Nodes with high degree (highly connected) are called hubs and hold together several nodes with lower degree. Networks displaying a degree distribution approximating a power-law, $P(k)\ k^{\gamma}$, where γ is degree exponent and indicates proportional to, are called scale-free networks. Scale-free networks are mainly dominated by hubs (the smaller the value of γ the more important is the role of the hubs in the network) and are intrinsically robust to random attacks but vulnerable to selected alterations. Scale-free networks are typically natural networks.

In biological terms

The degree allows an immediate evaluation of the regulatory relevance of the node. For instance, in signaling networks, proteins with very high degree are interacting with several other signaling proteins, thus suggesting a central regulatory role, that is they are likely to be regulatory hubs. For instance, signaling proteins encoded by oncogenes, such as HRAS, SRC or TP53, are hubs. Depending on the nature of the protein, the degree could indicate a central role in amplification (kinases), diversification and turnover (small GTPases), signaling module assembly (docking proteins), gene expression (transcription factors), etc. Signaling networks have typically a scale-free architecture.

Diameter (Δ_G)

$\Delta G =$ the maximal distance (shortest path) amongst all the distances calculated between each couple of vertexes in graph G. The diameter indicates how much distant are the two most distant nodes. It can be a first and simple general parameter of graph compactness, meaning with that the overall proximity between nodes. A high graph diameter indicates that the two nodes determining that diameter are very distant, implying little graph compactness. However, it is possible that two nodes are very distant, thus giving a high graph diameter, but several other nodes are not. Therefore, a graph could have high diameter and still being rather compact or have very compact regions. Thus, a high graph diameter can be misleading in term of evaluation of graph compactness. In contrast a low graph diameter is much more informative and reliable. Indeed, a low diameter surely indicates that all the nodes are in proximity and the graph is compact. In quantitative terms, high and low are better defined when compared to the total number of nodes in the graph. Thus, a low diameter of a very big graph (with hundreds of nodes) is much more meaningful in term of compactness than a low diameter of a small graph (with few nodes). Notably, the diameter enables to measure the development of a network in time.

In biological terms

The diameter, and thus the compactness, of a biological network, for instance a protein-signaling network, can be interpreted as the overall easiness of the proteins to communicate and/or influence their reciprocal function. It could be also a sign of functional convergence. Indeed, a big protein network with low diameter may suggest that the proteins within the network had a functional co-evolution. The diameter should be carefully weighted if the graph is not fully connected (that is, there are isolated nodes).

Average Distance (AvD_G)

$AvD_G =$ the average shortest path of a graph G, corresponding to the summa of all shortest paths between vertex couples divided for the total number of vertex couples. Often it is not an integer. As for the diameter, it can be a simple and general parameter of graph compactness, meaning with that the overall tendency of nodes to stay in proximity. Being an average, it can be somehow more informative than the diameter and can be also considered a general indicator of network navigability. A high average distance indicates that the nodes are distant (disperse), implying little graph compactness. In contrast a low average distance indicates that all the nodes are in proximity and the graph is compact. In quantitative terms, high and low are better defined when compared to the total number of nodes in the graph. Thus, a low average distance of a very big graph (with hundreds of nodes) is more meaningful in term of compactness than a low average distance of a small graph (with few nodes).

In biological terms

The average distance of a biological network, for instance a protein-signaling network, can be interpreted as the overall easiness of the proteins to communicate and/or influence their reciprocal function. It could be also a sign of functional convergence. Indeed, a big protein network with low average distance may suggest that the proteins within the network have the tendency to generate functional complexes and/or modules (although centrality indexes should be also calculated to support that indication).

Eccentricity ($C_{ecc}(v)$)

$$C_{ecc}(v) := \frac{1}{max\{dist(v,w) : w \in V\}}$$

The eccentricity is a node centrality index. The eccentricity of a node v is calculated by computing the shortest path between the node v and all other nodes in the graph, then the longest shortest path is chosen (let (v, K) where K is the most distance node from v). Once this path with length $dist(v, K)$ is identified, its reciprocal is calculated $(1/dist(v, K))$. By doing that, an eccentricity with higher value assumes a positive meaning in term of node proximity. Indeed, if the eccentricity of the node v is high, this means that all other nodes are in proximity. In contrast, if the eccentricity is low, this means that there is at least one node (and all its neighbors) that is far form node v. Of course, this does not exclude that several other nodes are much closer to node v. Thus, eccentricity is a more meaningful parameter if is high. Notably, high and low values are more significant when compared to the average eccentricity of the graph G calculated by averaging the eccentricity values of all nodes in the graph.

In biological terms

The eccentricity of a node in a biological network, for instance a protein-signaling network, can be interpreted as the easiness of a protein to be functionally reached by all other proteins in the network. Thus, a protein with high eccentricity, compared to the average eccentricity of the network, will be more easily influenced by the activity of other proteins (the protein is subject to a more stringent or complex regulation) or, conversely could easily influence several other proteins. In contrast, a low eccentricity, compared to the average eccentricity of the network, could indicate a marginal functional role (although this should be also evaluated with other parameters and contextualized to the network annotations).

Closeness ($C_{clo}(v)$)

$$C_{clo}(v) := \frac{1}{\sum_{w \in V} dist(v,w)}$$

The closeness is a node centrality index. The closeness of a node v is calculated by computing the shortest path between the node v and all other nodes in the graph, and then calculating the summa. Once this value is obtained, its reciprocal is calculated, so higher values assume a positive meaning in term of node proximity. Also here, high and low values are more meaningful when compared to the average closeness of the graph G calculated by averaging the closeness values of all nodes in the graph. Notably, high values of closeness should indicate that all other nodes are in proximity to node v. In contrast, low values of closeness should indicate that all other nodes are distant from node v. However, a high closeness value can be determined by the presence of few nodes very close to node v, with other much more distant, or by the fact that all nodes are generally very close to v. Likewise, a low closeness value can be determined by the presence of few nodes very distant from node v, with other much closer, or by the fact that all nodes are generally distant from v. Thus, the closeness value should be considered as an average tendency to node proximity or isolation, not really informative on the specific nature of the individual node couples. The closeness should be always compared to the eccentricity: a node with high eccentricity + high closeness is very likely to be central in the graph.

In biological terms

The closeness of a node in a biological network, for instance a protein-signaling network, can be interpreted as the probability of a protein to be functionally relevant for several other proteins, but with the possibility to be irrelevant for few other proteins. Thus, a protein with high closeness, compared to the average closeness of the network, will be easily central to the regulation of other proteins but with some proteins not influenced by its activity. Notably, in biological networks could be also of interest to analyze proteins with low closeness, compared to the average closeness of the network, as these proteins, although less relevant for that specific network, are possibly behaving as intersecting boundaries with other networks. Accordingly, a signaling network with a very high average closeness is more likely organizing functional units or modules, whereas a signaling network with very low average closeness will behave more likely as an open cluster of proteins connecting different regulatory modules.

Radiality ($C_{rad}(v)$)

$$C_{rad}(v) := \frac{\sum_{w \in V}(\Delta_G + 1 - dist(v,w))}{n-1}$$

The radiality is a node centrality index. The radiality of a node v is calculated by computing the shortest path between the node v and all other nodes in the graph. The value of each path is then subtracted by the value of the diameter +1 ($\Delta G + 1$) and the resulting values are summated. Finally, the obtained value is divided for the number of nodes -1 ($n-1$). Basically, as the diameter is the maximal possible distance between nodes, subtracting systematically from the diameter the shortest paths between the node v and its neighbors will give

high values if the paths are short and low values if the paths are long. Overall, if the radiality is high this means that, with respect to the diameter, the node is generally closer to the other nodes, whereas, if the radiality is low, this means that the node is peripheral. Also here, high and low values are more meaningful when compared to the average radiality of the graph G calculated by averaging the radiality values of all nodes in the graph. As for the closeness, the radiality value should be considered as an average tendency to node proximity or isolation, not definitively informative on the centrality of the individual node. The radiality should be always compared to the closeness and to the eccentricity: a node with high eccentricity + high closeness+ high radiality is a consistent indication of a high central position in the graph.

In biological terms

The radiality of a node in a biological network, for instance a protein-signaling network, can be interpreted as the probability of a protein to be functionally relevant for several other proteins, but with the possibility to be irrelevant for few other proteins. Thus, a protein with high radiality, compared to the average radiality of the network, will be easily central to the regulation of other proteins but with some proteins not influenced by its activity. Notably, in biological networks could be also of interest to analyze proteins with low radiality, compared to the average radiality of the network, as these proteins, although less relevant for that specific network, are possibly behaving as intersecting boundaries with other networks. Accordingly, a signaling network with a very high average radiality is more likely organizing functional units or modules, whereas a signaling network with very low average radiality will behave more likely as an open cluster of proteins connecting different regulatory modules. All these interpretations should be accompanied to the contemporary evaluation of eccentricity and closeness.

Centroid value ($C_{cen}(v)$)

$$C_{cen}(v) := min\{f(v,w) : w \in V\{v\}\}$$

Where $f(v,w) := \gamma_v(w) - \gamma_w(v)$, and $\gamma_v(w)$ is the number of vertex closer to v than to w. The centroid value is the most complex node centrality index. It is computed by focusing the calculus on couples of nodes (v,w) and systematically counting the nodes that are closer (in term of shortest path) to v or to w. The calculus proceeds by comparing the node distance from other nodes with the distance of all other nodes from the others, such that a high centroid value indicates that a node v is much closer to other nodes. Thus, the centroid value provides a centrality index always weighted with the values of all other nodes in the graph. Indeed, the node with the highest centroid value is also the node with the highest number of neighbors (not only first) if compared with all other nodes. In other terms, a node v with the highest centroid value is the node with the highest number of neighbors separated by the shortest path to v. The centroid value suggests that a specific node has a central position within a graph

region characterized by a high density of interacting nodes. Also here, high and low values are more meaningful when compared to the average centrality value of the graph G calculated by averaging the centrality values of all nodes in the graph.

In biological terms

The centrality value of a node in a biological network, for instance a protein-signaling network, can be interpreted as the probability of a protein to be functionally capable of organizing discrete protein clusters or modules. Thus, a protein with high centroid value, compared to the average centroid value of the network, will be possibly involved in coordinating the activity of other highly connected proteins, altogether devoted to the regulation of a specific cell activity (for instance, cell adhesion, gene expression, proliferation etc.). Accordingly, a signaling network with a very high average centroid value is more likely organizing functional units or modules, whereas a signaling network with very low average centroid value will behave more likely as an open cluster of proteins connecting different regulatory modules. It can be useful to compare the centroid value to other algorithms detecting dense regions in a graph, indicating protein clusters, such as, for instance, MCODE.

Stress ($C_{str}(v)$)

$$C_{str}(v) := \sum_{s \neq v \in V} \sum_{t \neq v \in V} \sigma_{st}(v)$$

The stress is a node centrality index. Stress is calculated by measuring the number of shortest paths passing through a node. To calculate the stress of a node v, all shortest paths in a graph G are calculated and then the number of shortest paths passing through v is counted. A stressed node is a node traversed by a high number of shortest paths. Notably, and importantly, a high stress values does not automatically implies that the node v is critical to maintain the connection between nodes whose paths are passing through it. Indeed, it is possible that two nodes are connected by means of other shortest paths not passing through the node v. Also here, high and low values are more meaningful when compared to the average stress value of the graph G calculated by averaging the stress values of all nodes in the graph.

In biological terms

The stress of a node in a biological network, for instance a protein-signaling network, can indicate the relevance of a protein as functionally capable of holding together communicating nodes. The higher the value the higher the relevance of the protein in connecting regulatory molecules. Due to the nature of this centrality, it is possible that the stress simply indicates a molecule heavily involved in cellular processes but not relevant to maintain the communication between other proteins.

S.-P. Betweenness ($C_{spb}(v)$)

$$C_{spb}(v) := \sum_{s \neq v \in V} \sum_{t \neq v \in V} \delta_{st}(v)$$

where

$$\delta_{st}(v) := \frac{\sigma_{st}(v)}{\sigma_{st}}$$

The S.-P. Betweenness is a node centrality index. It is similar to the stress but provides a more elaborated and informative centrality index. It is calculated considering couples of nodes $(v1, v2)$ and counting the number of shortest paths linking $v1$ and $v2$ and passing through a node n. Then, the value is related to the total number of shortest paths linking $v1$ and $v2$. Thus, a node can be traversed by only one path linking $v1$ and $v2$, but if this path is the only connecting $v1$ and $v2$ the node n will score a higher betweenness value (in the stress computation would have had a low score). Thus, a high S.-P. Betweenness score means that the node, for certain paths, is crucial to maintain node connections. Notably, to know the number of paths for which the node is critical it is necessary to look at the stress. Thus, stress and S.-P. Betweenness can be used to gain complementary information. Further information could be gained by referring the S.-P. Betweenness to node couples, thus quantifying the importance of a node for two connected nodes. Also here, high and low values are more meaningful when compared to the average S.-P. Betweenness value of the graph G calculated by averaging the S.-P. Betweenness values of all nodes in the graph.

In biological terms

The S.-P. Betweenness of a node in a biological network, for instance a protein-signaling network, can indicate the relevance of a protein as functionally capable of holding together communicating proteins. The higher the value the higher the relevance of the protein as organizing regulatory molecule. The S.-P. Betweenness of a protein effectively indicates the capability of a protein to bring in communication distant proteins. In signaling modules, proteins with high S.-P. Betweenness are likely crucial to maintain functionally and coherence of signaling mechanisms.

Eigenvector Centrality

The Eigenvector centrality is a node centrality index. It assigns relative scores to all nodes in the network based on the concept that connections to high-scoring nodes contribute more to the score of the node in question than equal connections to low-scoring nodes. The definition is a recursive definition:an high eigenvector value means that a node has several neighbors with high eigenvector value. So, the eigenvector centrality can be viewed as a sort of weighted degree, where not only the number of the neighbors is important but also the score (the eigenvector itself) of the neighbors. A High Eigenvector centrality indicates that

the node is being visited a lot while traversing and is well-connected. A variant of the Eigenvector algorithm is used by Google's Page Rank algorithm.

In biological terms

Similarly to the degree, Eigenvector allows an immediate evaluation of the regulatory relevance of the node. A protein with a very high Eigenvector is a protein interacting with several important proteins, thus suggesting a central regulatory role. A protein with low Eigenvector, can be considered a peripheral protein, interacting with few and not central proteins.

Bridging Centrality

The Bridging Centrality of a node is the product of the Bridging Coefficient (BC) and the Betweenness centrality (Btw). The Bridging Coefficient of a node is high if the node has neighbors with high degree. This means that the bridging coefficient is high if the node has highly connected first neighbors. Since the Bridging Centrality is equals to BC*Btw, this means that a node has high Bridging Centrality if it is a connecting node (due to Btw component) of high degree nodes (due to BC component). This can suggest that the node is a connecting node between clusters or between dense region of the network.

In biological terms

The Btw component of the Bridging centrality, suggests that in a biological network such as, for example, a protein-protein interaction network, a protein with high Bridging Centrality value is functionally capable of holding together communicating proteins. The BC components may also suggest that such communicating proteins are regulatory proteins since they interact with many other proteins. Thus, a protein with high Bridging centrality is a protein possibly bringing in communication sets of regulatory proteins. In a context of directed networks, a high value of Bridging Centrality may indicate that a protein is regulated by many proteins at once or that is regulating the overall architecture of many clusters at once.

Eigenvector centrality

Eigenvector centrality is a node centrality index. It assigns relative scores to all nodes in a network based on the concept that connections to high-scoring nodes contribute to the score of a specific node more than equal connections to low-scoring nodes. The definition is recursive: an high Eigenvector value means that a node has many neighbors with high Eigenvector value. So, the Eigenvector centrality can be viewed as a sort of weighted degree, where not only the number of the neighbors is important but also the score (the Eigenvector itself) of the neighbors. A high Eigenvector centrality indicates that a node is being visited a lot while traversing and is well-connected. A variant of the Eigenvector algorithm is used by Google's Page Rank algorithm.

In biological terms

Eigenvector allows an immediate, rather informative, evaluation of the regulatory relevance of the node. For instance, a protein with a very high Eigenvector is a protein interacting with several important proteins (regulating them or being regulated by them), thus suggesting a central super-regulatory role or a critical target of a regulatory pathway. In contrast, a protein with low Eigenvector, can be considered a peripheral protein, interacting with few and not central proteins. In a context of directed networks, calculation of Eigenvector could be useful to estimate the output of the pharmacological modulation of a specific protein. Indeed, pharmacological modulation (for instance inhibition) of a high Eigenvector protein can generate broad biological effects but potentially characterized by consistent side-effects, whereas, pharmacological modulation of a low Eigenvector protein could generate limited, more focused, effects with minimal side-effects. Thus, depending of the context, the Eigenvector could potentially help to identify best treatments for diseases.

Edge Betweenness

The Edge betweenness centrality is an edge centrality index. It is the edge version of the node betweenness centrality. Given an edge E, it is calculated considering couples of nodes (v1, v2) and counting the number of shortest paths linking v1 and v2 and passing through the edge E. Then, the value is related to the total number of shortest paths linking v1 and v2. Thus, an edge can be traversed by only one path linking v1 and v2, but if this path is the only connecting v1 and v2 the edge will score a higher betweenness value. Thus, a high S.-P. Betweenness score means that the edge, for certain paths, is crucial to maintain node connections. Edge betweenness scores are reported in the edge attribute table.

In biological terms

Since the Edge-Betweenness is an edge score of centrality, with the edge indicating the functional relationship between two nodes (for instance a kinase-triggered phosphorylation), in a biological network such as, for instance, a protein-signaling network, computation of this centrality moves the focus from node relevance to the biochemical process relevance, thus indicating that a specific biochemical reaction (or any other type of functional relationship) has a central role in the network functional organization. The higher the value the higher the relevance of the interaction as organizing regulatory process. In signaling modules, interactions with high E-Betweenness are likely prevalent to maintain functionally and coherence of signaling mechanisms. In a metabolic network, a high Edge Betweenness value is related to the relevance of a reaction in energy and mass processing. Overall, Edge Betweenness quantify the capability of a certain reaction to be a information (signaling) or mass (metabolic) transit reaction, a sort of obligatory passage point for the reactions modeling the network.
