# Supplementary material for: Biological network analysis with CentiScaPe: centralities and experimental dataset integration
Source: F1000Res. 2015 Jul 7;3:139. Originally published 2014 Jul 1. [Version 2] doi: 10.12688/f1000research.4477.2 (PMC4647866; doi:10.12688/f1000research.4477.2)
Supplement: Peer review history

# Peer review: Referee response for version 2

**DOI:** 10.5256/f1000research.6690.r9389

**Published:** 2015-07-23

**Authors:** Ankush Sharma

**Affiliations:** 1 Laboratory for Genomics,Transcriptomics and Proteomics, High Performance Computing and Networking Institute, National Research Council, Naples, Italy

The Authors have addressed the concerns raised by reviewers and  made neccesary changes in manuscript. The manuscript is fully approved for indexing.

I have read this submission. I believe that I have an appropriate level of expertise to confirm that it is of an acceptable scientific standard.

# Peer review: Referee response for version 2

**DOI:** 10.5256/f1000research.6690.r5339

**Published:** 2015-07-08

**Authors:** Ferenc Jordan

**Affiliations:** 1 Centre for Computational and Systems Biology, University of Trento, Trento, Italy

The Authors have made the necessary changes so it is now fully approved by me.

I have read this submission. I believe that I have an appropriate level of expertise to confirm that it is of an acceptable scientific standard.

# Peer review: Referee response for version 1

**DOI:** 10.5256/f1000research.4788.r5341

**Published:** 2014-07-11

**Authors:** Ankush Sharma

**Affiliations:** 1 Laboratory for Genomics,Transcriptomics and Proteomics, High Performance Computing and Networking Institute, National Research Council, Naples, Italy

In this research article, the authors describe the CentiScaPe app for Cytoscape (and its web version FastCentiScaPe) for computing the several centrality parameters in networks, analyzing the elemental level importance of each node in the network, based on network topology. The addition of centralities parameters, especially Bridging Centrality and Eigenvector centrality, appears to be useful to improve metrics, to infer the informational flow across overlapping modules, and as a measure of the influence of a node in a network. The article is well written with an appropriate title and an abstract which provides sufficient details. The description of the purpose, implementation and usage of CentiScaPe app are informative and detailed for the audience.

**Minor comments:**

1. The example of calculating stress in the network of oncogenes in the manuscript should comply with experimental functional relevance, e.g. a decrease in bridging centrality is observed for key intermodular nodes upon heat shock response or oxidative stress, partially disintegrating overlapping modules into local communities which alters the centrality statistics of the nodes ^1^^,^^2^. It would be good to see such implementation in the example showing applicability of CentiScaPe.
2. I suggest the authors have a small example of the analysis comparing connected and disconnected networks, to support computation of centralities for networks with a disconnected component.
3. It would be better to implement a user-friendly exploration of results and user registration feature instead of email authorization, in order to avoid delays in using the web version FastCentiScaPe.
4. Correction in the name of built-in tool i.e. NetworkAnalyzer , instead of "Analyze Network" tool.
5. Usage of appropriate words such as “more comprehensive” in place of “more complete” in introduction section.

I have read this submission. I believe that I have an appropriate level of expertise to confirm that it is of an acceptable scientific standard, however I have significant reservations, as outlined above.

## References

[1] LehtinenSMarsellachFXCodlinSSchmidtAClément-ZizaMBeyerABahlerJOregnoCPancaldiV: Stress induces remodelling of yeast interaction and co-expression networks. *Mol Biosyst*.2013;9(7) : 139 1697-1707 10.1039/c3mb25548d Reference source 23471351

[2] MihalikACsermelyP: Heat shock partially dissociates the overlapping modules of the yeast protein-protein interaction network: a systems level model of adaptation. *PLoS Comput Biol*.2011;7(10) : 139 10.1371/journal.pcbi.1002187 Reference source 22022244PMC3192799

# Peer review: Referee response for version 1

**DOI:** 10.5256/f1000research.4788.r5358

**Published:** 2014-07-07

**Authors:** Ferenc Jordan

**Affiliations:** 1 Centre for Computational and Systems Biology, University of Trento, Trento, Italy

Analysing several centrality measures in parallel is important and there is a need for this, however, it would be even nicer to compare them (see http://www.sciencedirect.com/science/article/pii/S0304380007001184 ) and discuss better their biological relevance (i.e. what is Eigenvector centrality good for?). Central nodes can be of key regulator function but this is not a result, this is still a hypothesis. It should be mentioned briefly that either simulations or lab experiments should reinforce these findings. For infinite distances, using the reciprocal distance matrix has already been suggested as an elegant solution (see http://link.springer.com/article/10.1007/BF01164642#page-1). It should be clarified a bit more that a large weight can be considered as a short or as a long path, depending on its biological meaning. In Figure 1, the grid layout algorithm could be replaced by some better one, I think. Finally, a quick English check would be welcome.

I have read this submission. I believe that I have an appropriate level of expertise to confirm that it is of an acceptable scientific standard, however I have significant reservations, as outlined above.
